# Quantitative detection of circulating MT-ND1 as a potential biomarker for colorectal cancer

**DOI:** 10.17305/bjbms.2021.5576

**Published:** 2021-10

**Authors:** Yichun Xu, Jiajing Zhou, Qing Yuan, Jun Su, Qian Li, Xiaoliang Lu, Liwen Zhang, Zhai Cai, Junsong Han

**Affiliations:** 1 Shanghai National Engineering Research Center for Biochip at Shanghai and Shanghai Biochip Limited Corporation, Shanghai, China; 2 Department of Pathology, Shanghai Tongji Hospital, Tongji Hospital Affiliated to Tongji University, Shanghai, China; 3 Department of General Surgery, Zhujiang Hospital, Southern Medical University, Guangzhou, China

**Keywords:** MT-ND1, colorectal cancer, mtDNA, cfDNA, biomarker

## Abstract

Liquid biopsy represents a diagnostic and monitoring tool and the circulating cell-free mitochondrial DNA (mtDNA) plays a vital role in tumor diagnosis and dynamic assessment. Colorectal cancer (CRC) is one of the most common fatal cancers worldwide. Mitochondrially encoded NADH dehydrogenase subunit 1 (MT-ND1) encodes the biggest subunit of respiratory complex I of mtDNA, and mutations in the MT-ND1 are common in CRC. We sought to determine if mutations in circulating MT-ND1 could be a potential biomarker for colorectal cancer. In this study, twenty-two CRC patients at Zhujiang Hospital were included. We mainly used droplet digital PCR to determine the mutation status of MT-ND1, combined with clinical data. In the experiment in vivo, cell-free mtDNA generally presented high concordance with tumor tissues. By quantitative PCR, the MT-ND1 content of plasma in CRC patients was significantly higher than that in healthy individuals (58.01 vs. 0.64, p=0.027). The detection of circulating MT-ND1 content and variants (m.3606 A>G, m.3970 C>T, m.4071 C>T, m.4086 C>T) in cfDNA showed a good correlation with predicted tumor response and progression to chemotherapy. In conclusion, the content and variants of circulating MT-ND1 may become a versatile tool for the diagnosis and monitoring of colorectal cancer.

## INTRODUCTION

Colorectal cancer (CRC) is the third most common fatal cancer worldwide [1]. The overall 5-year survival of patients with CRC remains very low. Effective biomarkers for the early detection, diagnosis, prognosis and monitoring of CRC are urgently needed.

Nowadays, the cell-free mitochondrial DNA (mtDNA) has been received much attention in cancer diagnosis and prognosis. Compared to nuclear DNA (nDNA), mtDNA is very vulnerable to mutations, and the copy number of mtDNA per cell may increase to compensate for DNA damage [2]. The increase of mtDNA copy number per cell may lead to compensation of mtDNA damage or mitochondrial dysfunction [3]. Elevated mtDNA copy number in plasma or serum has been proven to associate with an increased risk of several malignancies including non-Hodgkin lymphoma [4], lung cancer [5] and pancreatic cancer [6]. Complex I harboring subunits is the largest component of the respiratory chain, which catalyzes the transfer of electrons from NADH to flavin mononucleotide and then to ubiquinone [7]. Mitochondrially encoded NADH dehydrogenase subunit 1 (MT-ND1), encodes one of the seven subunits of respiratory complex I and is involved in the first step of the electron transport chain of oxidative phosphorylation (OXPHOS). Mutations in MT-ND1 might cause an alteration of the electron transport components and damage the normal electron flow, leading to an increase of superoxidase radical generation and increase oxidative stress in various types of cancer cells [8]. Some variants of MT-ND1, including A4164G, A4123G, T3394C, A3434G and C3497T, exist in the colorectal tumor tissues comparing to the corresponding adjacent tissue [9]. In recent years, liquid biopsy is a useful diagnosis and monitoring tool, which has been gained extensive attention as a noninvasive detection tool. However, it is not clear whether circulating MT-ND1 could be used as an effective biomarker using liquid biopsy strategies for CRC.

In this study, we investigated the concordance of MD-ND1 mutations between cfDNA and corresponding tumor tissues and analyze the character of cell-free mtDNA in longitudinal cases. We aimed to find out whether the content and variants of circulating MT-ND1 could be a potential biomarker for colorectal cancer.

## MATERIALS AND METHODS

### Detection of MT-ND1 mutation for CRC cell lines *in vitro* and *in vivo*

The human colorectal cancer cell lines LoVo and HT-29 were purchased from Cell Bank of Type Culture Collection of Chinese Academy of Sciences (Shanghai, China). All the cells were cultured in monolayer using RPMI 1640 medium supplemented with 10% (v/v) fetal bovine serum (FBS) (Gibco, USA). Cells were maintained in a humidified atmosphere of 5% CO_2_ and 95% air at 37°C.

The whole genome DNA of cell lines was extracted from the two cell lines using Genomic DNA Extraction Kit (Shanghai Biochip Co., Ltd., China), according to the manufacturer’s protocol. The DNA concentration was measured with NanoDrop One spectrophotometer (Thermo Fisher Scientific, Inc.) and the quality of DNA was monitored by electrophoresis.

The primers used for PCR were listed in Table 1 and were synthesized by Sangon Biotech (Shanghai) Co., Ltd.. The reaction system included 1μL DNA (15–20 ng), 0.2 μLof 5 U/μL rTaq PCR Polymerase, 0.3 μL of 10 mM dNTP, 1.2 μL of 25 mM Mg^2+^, 1 μL of both primers and 8.8 μL ddH_2_O. The progress of amplification was composed of an initial polymerase activation at 95°C for 5 min, followed by 15 cycles at 95°C for 30 s, with annealing at 65°C (−1°C/cycle) for 30 s and 72°C for 45 s, and20 cycles at 95°C for 30 s, with annealing at 50°C for 30 s and 72°C for 45 s. The prior amplification was performed on T100 Thermal Cycler (BioRad). The PCR products were sequenced using ABI3730 DNA sequencer (Applied Biosystems, Foster City, CA, USA).

**TABLE 1:**
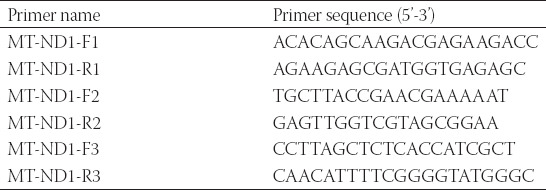
The primer sequences of MT-ND1 for Sanger's sequencing

To validate the concordance of mitochondrial mutations between cfDNA and its corresponding tumor, the vivo experiment was performed. We transplanted LoVo and HT-29 cells (5×10^6^) by subcutaneous injection into BALB/c-nu/nu male mice (n=6, Shanghai Slac Laboratory Animal). Meanwhile, another six mice were injected with PBS. Environmental conditions were a temperature of 22 ± 2°C, humidity of 50 ± 10%, 12 h light and 12 h dark cycle with lights on at 07:00 and off at 19:00. These mice were sacrificed after 6 weeks. The tumors were surgical excised, and the genomic DNA was extracted from the tumors for PCR as described above. The blood samples were collected in EDTA tubes by heart puncture, processed into plasma within 30 min (2,000g for 15 min at 4°C). The cell-free circulating DNA (cfDNA) of plasma was extracted by QIAamp Circulating Nucleic Acid Kit (Qiagen), according to the manufacturer’s protocol. The DNA concentrations were determined by Qubit dsDNA HS Assay Kit (Life Technologies) and cfDNA quality was evaluated with the Agilent 4200 Bioanalyzer.

Using the primers and probes in Table 2, droplet digital PCR (ddPCR) was performed with QX200 Droplet Digital PCR system (BioRad) to detect the mutation rate of MT-ND1 in plasma and related tumor tissue. A final volume of 20 μL reaction mixture comprised 10 μL 2 × ddPCR Supermix for Probes, 1.8 μL of both primers (10 μM), 0.5 μL of both probes (10 μM), 5.4 μL of ddH_2_O and template. The progress of amplification was composed of one cycle at 95°C for 10 min, 40 cycles of 94 °C for 30 s and 55 °C for 1 min, followed by 10 °C hold. We analyzed the results using QuantaSoft (ver 1.6.6) software that accompanied the QX200 reader. In our analyses, MT-ND1 mutation-specific signals are generated in the FAM channel while the MT-ND1 wildtype signals are in the VIC channel. We calculated poisson concentrations using QuantaSoft, and the MT-ND1 mutation rate was calculated from generated Poisson concentrations as follows: % Mut = [FAM]/[FAM+VIC]×100%.

**TABLE 2:**
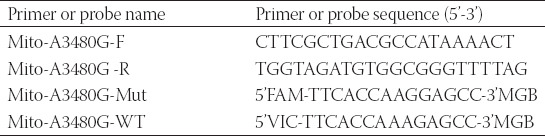
The primer and probe sequences of MT-ND1 for ddPCR detection

### Patient cohort

Twenty-two CRC patients were seen at Zhujiang Hospital, and the patient baseline characteristics were shown in Table S1. Eighteen of these patients had tumor tissues, and the rest of the patients had blood samples of the whole treatment process including excision and chemotherapy course. Four healthy individuals were also included in the study. The study protocol has been approved by the Ethics Committee of the Zhujiang Hospital (approval number: 2014-PTWK-1010). All study participants provide their informed consent before inclusion into the study.

### Mutation detection of MT-ND1 for CRC patients

Eighteen CRC patients with malignancy-free margins were included. Their tumor tissues were utilized to extract genomic DNA. The extraction of genome DNA, the measurement of DNA concentration, the monitoring of DNA quality, the primers and the PCR condition were described above. In order to exclude germline variants, we collected mitochondrial germline variants of cancers from the database of The Cancer Genome Atlas (TCGA) and published articles. Then we compared the hotspots of CRC patients with reported mitochondrial germline variants of cancers, and finally identified the somatic mutations.

### Detection of MT-ND1 content for CRC patients

Four CRC patients and four healthy individuals were included. 5 mL of blood samples were collected in EDTA tubes, processed into plasma within 30 min (2,000g for 15 min at 4°C). The extraction and quality assessment of the cfDNA from the plasma were described above. The primers and the PCR condition used were described in Table 3. The Student’s t-test was used as a statistical analysis for the content of MT-ND1.

**TABLE 3:**
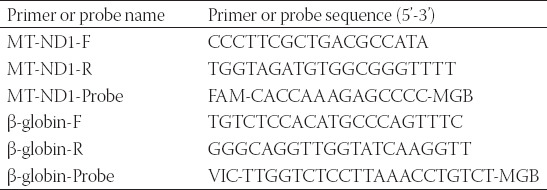
The primers and probes for detection of MT-ND1 content

### Bioanalysis in cfDNA of longitudinal cases

Four CRC patients with malignancy-free margins were included. The time points for collecting the blood samples included before surgery (Pre), three days after the operation (Post), the first course of chemotherapy (1^st^); the third course of chemotherapy (3^rd^); the fifth course of chemotherapy (5^th^). 5 mL of blood samples for each time point were collected in EDTA tubes, processed into plasma within 30 min (2,000g for 15 min at 4°C). A part of the plasma was used for mutational analysis. The extraction and quality assessment of the cfDNA from the plasma were described as above.

To determinate the correlation between the character of cfDNA and the relapse of CRC, the abundance and completeness of cfDNA on each stage of patients with CRC were evaluated. The abundance of cfDNA was just as the result of Qubit detection. The completeness of cfDNA was evaluated by qPCR, using the ratio of 247 bp ALU and 115 bp ALU (Table 4). A final volume of 20 μL reaction mixture comprised 2× Master Mix (Life Technologies), 0.5 μL for each primer (10 μM), 0.3 μL for each probe (10 μM), and the rest for template and ddH_2_O. The progress of amplification was composed of one cycle at 95°C for 10 min and 40 cycles at 95°C for 15 s, 60°C for 1 min.

**TABLE 4:**
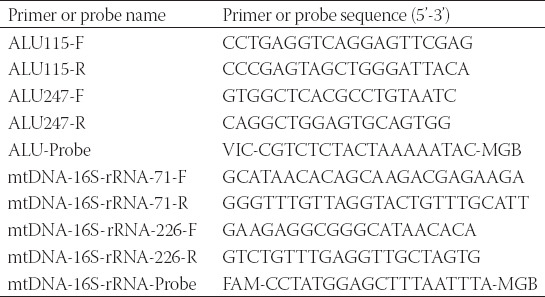
The primers for abundance and completeness of cfDNA and cell free mtDNA detection

To determine the correlation between the character of cell-free mtDNA and the relapse of CRC, the abundance and completeness of cell-free mtDNA in each stage of patients with CRC were evaluated. The abundance of cell-free mtDNA was determined by qPCR for the quantification of MT-ND1 using the primers and probes in Table 3 [10]. The completeness of cell-free mtDNA was evaluated by qPCR, using the ratio of 226 bp 16S and 71 bp 16S (Table 4).

Based on the sequencing result of the eighteen CRC patients, we had found four hotspots of MT-ND1. In this part, we would like to find out the correlation between the MT-ND1 mutational load and the stage of CRC. Then ddPCR was performed using the primers and probes in Table 5. The reaction system, condition and analysis of ddPCR were described above. The average mutation load of MT-ND1 for an individual was the average mutation percentage in four genes.

**TABLE 5:**
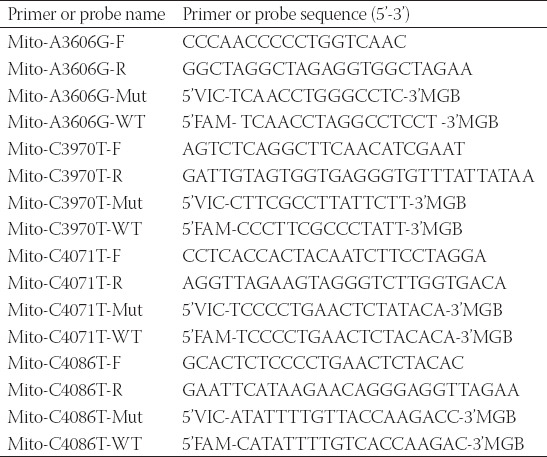
The primer and probe sequences of MT-ND1 for ddPCR detection


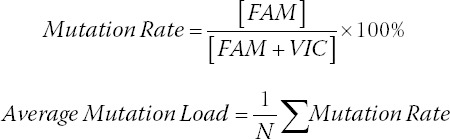


where N is the total number of hotspots, and mutation rate is the ratio of positive concentration in FAM passage and total positive concentration.

### Analysis of the levels of carcinoembryonic antigen (CEA), carbohydrate antigen 19-9 (CA19-9) and carbohydrate antigen 72-4 (CA72-4) in plasma of longitudinal cases

Blood was collected in a tube without anticoagulant, and the supernatant was collected after standing for 30 min. Then serum samples were obtained after centrifuging at 800 g and 16,000g respectively. CEA, CA72-4 and CA19-9 were all performed by Cobas E601 (Roche Diagnostics) in chemiluminescent immunoassays according to the manufacturer’s instructions. The normal reference values of the three serum tumor markers are CEA 0-3.4 ng/mL, CA72-4 0-9.8 U/mL and CA19-9 0-39.0 U/mL. Exceeding the upper limit of the normal threshold is considered positive.

## RESULTS

### The concordance of MD-ND1 mutations between cfDNA and corresponding tumor

**FIGURE 1:**
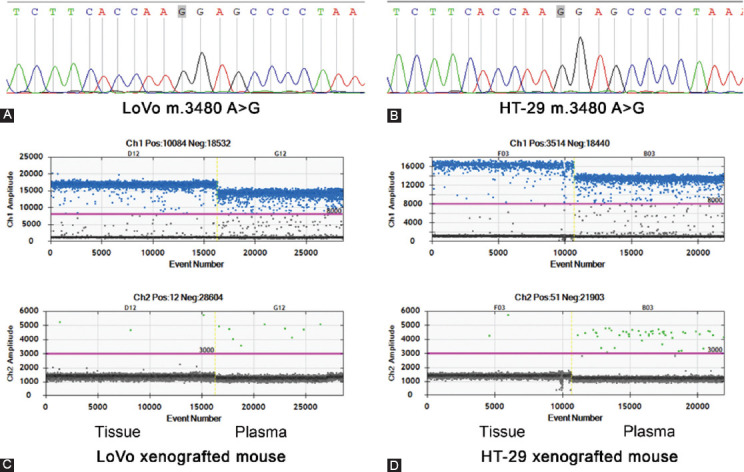
The concordance of MD-ND1 mutations between cfDNA and corresponding tumor (A) The cell line of LoVo showed mutant type for m.3480 A>G in MT-ND1. (B) The cell line of HT-29 showed mutant type for m.3480 A>G in MT-ND1. (C) 1-D plot showed the m.3480 A>G status in tumor tissue and plasma cfDNA of LoVo xenografted mouse. (D) 1-D plot showed the m.3480 A>G status in tumor tissue and plasma cfDNA of HT-29 xenografted mouse.

From the result of Sanger sequencing, we found a mutation site (m.3480A>G) in both human colorectal cancer cell lines of LoVo and HT-29 (Figure 1A and 1B). Then the transplanted tumor mice model was used. Both cfDNA and the genome DNA of tumor tissues were detected by ddPCR using the primers and probes for m.3480A>G. Due to the limited volume of plasma, we randomly mixed two plasma samples into one. Correspondingly, the genome DNA samples from the tumor tissues were also mixed. Finally, there were three plasma samples and three genome DNA samples in each group. The mut-MT-ND1 in both plasma and tumor tissue of LoVo and HT-29 xenografted mice were detected. As shown in Figure 1C and 1D, the concordance of mutation rate was similar. In the xenografted mice of HT-29, the average mutation rate of tumor tissues was 99.94%, while the average mutation rate of plasma was 99.73% (Table 6). In the xenografted mice of LoVo, the average mutation rate of tumor tissues was 99.80%, while the average mutation rate of plasma was 95.97% (Table 6). Meanwhile, the control mice did not have mut-MT-ND1. The result indicated that the concordance of MT-ND1 mutations between cfDNA and corresponding tumor was high.

**TABLE 6:**
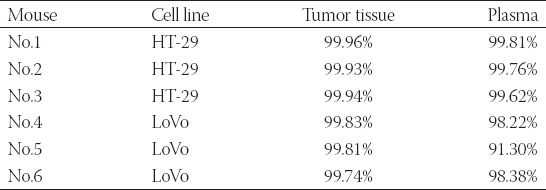
Comparison of m.3480A>G mutation rate between tumor tissue and plasma

### The mutation status of MT-ND1 for CRC patients

**FIGURE 2:**
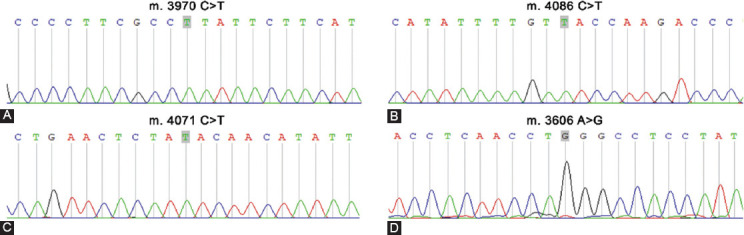
Mutations in MT-ND1 gene found in colorectal cancer. (A) The sequence data for mutant type of m. 3970 C>T. (B) The sequence data for mutant type of m. 4086 C>T. (C) The sequence data for mutant type of m. 4071 C>T. (D) The sequence data for mutant type of m. 3606 A>G.

Eighteen CRC patients with malignancy-free margins were included. The genomic DNA of tumor tissues was used to detect the mutation of MT-ND1. The result for mutational spots in MT-ND1 of eighteen CRC patients was shown in Table 7. Four mitochondrial mutational hot spots were detected in three or more cases. The proportion of the top four hot spots for m.3606 A>G, m.3970 C>T, m.4071 C>T and m.4086 C>T in CRC patients were 27.8% (5/18), 38.9% (7/18), 22.2% (4/18) and 27.8% (5/18) respectively (Figure 2). We also founded 44.4% (8/18) of the cases harbored two mitochondrial mutational hot spots. We collected mitochondrial germline variants of cancers from the database of TCGA and published articles (Table 8) [11]. The four hotspots we identified had not been included in the collected mitochondrial germline variants, and maybe these sites were somatic mutations.

**TABLE 7:**
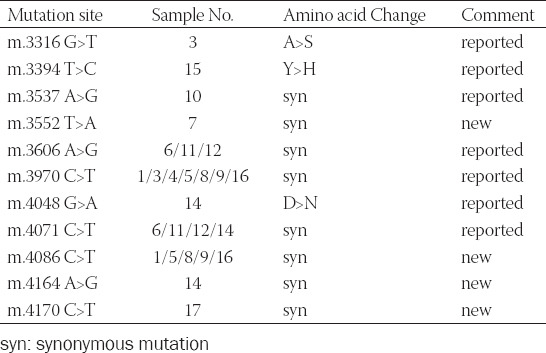
The result for mutational spots in MT-ND1 of CRC patients (n=18)

**TABLE 8:**
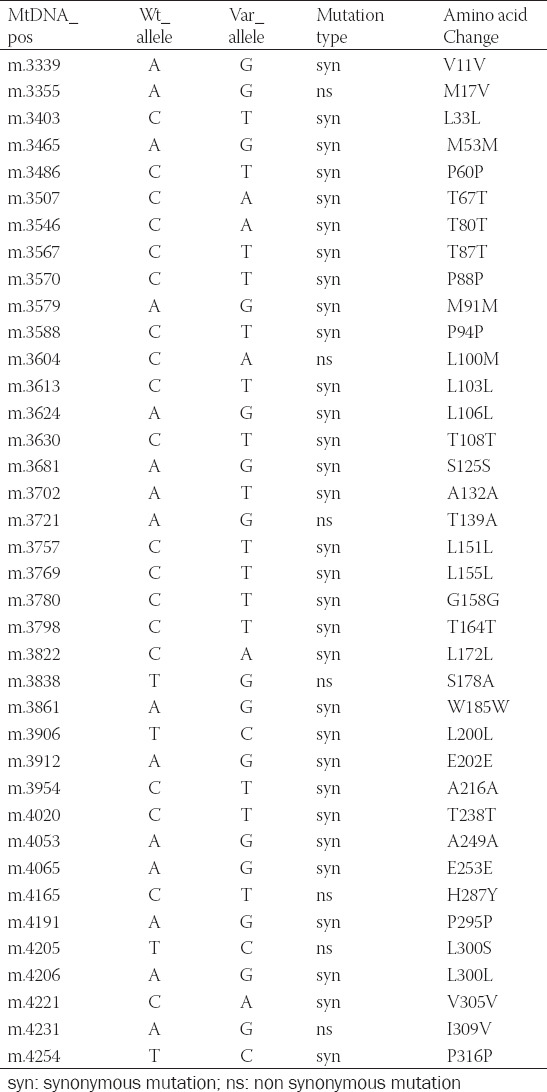
Germline mutations of MT-ND1 in TCGA database and published articles^11^

### Clinical characteristics of four CRC patients

The characteristics of four CRC patients, including diagnosis, TNM stage, differentiation, treatment and relapse, were reported in Table 9. Briefly, all the four patients were diagnosed as CRC with no metastasis. Three CRC patients are in the middle degree while only one CRC patient in the high degree. The first-line treatment for all the patients was Oxaliplatin combined Capecitabine. Three CRC patients showed clinical relapse, whereas one showed no sign of relapse.

**TABLE 9:**
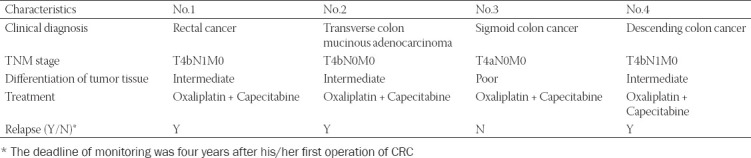
Clinical characteristics of four CRC patients

### The dynamic changes of cfDNA in plasma of longitudinal cases

**FIGURE 3:**
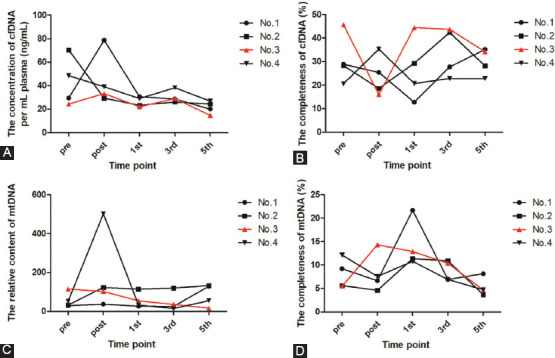
The dynamic changes of cfDNA and cell free mtDNA in plasma of longitudinal cases (A) The concentration of cfDNA in plasma of longitudinal cases. (B) The completeness of cfDNA in plasma of longitudinal cases. (C) The relative content of cell free mtDNA in plasma of longitudinal cases. (D) The completeness of cell free mtDNA in plasma of longitudinal cases.

To determine the correlation between the character of cfDNA in the treatment and the relapse of CRC, the abundance and completeness of cfDNA at each time point in the whole treatment were evaluated. As shown in Figure 3A, the level of cfDNA abundance increased after the surgery compared to the level before surgery, and then the level of cfDNA abundance decrease in most cases. Then we found that it was irregular for the dynamic changes in completeness of cfDNA (Figure 3B). Therefore, it seemed that there was no correlation between the character of cfDNA and prognosis of CRC. To determine the correlation between the character of cell-free mtDNA and the relapse of CRC, the abundance and completeness of cell-free mtDNA at each time point in the whole treatment were evaluated. We found that the level of mtDNA content continuously decreased only in No.3 patient with a good prognosis, while the changes were irregular in the other three patients (Figure 3C). In most cases, the level of mtDNA completeness decreased after the surgery comparing to the level before surgery, and then the level of mtDNA content in most cases also shown an irregular status. Interestingly, we found that the level of mtDNA completeness dramatically increased after surgery in No.3 patient with a good prognosis, and then continued to decline in the process of chemotherapy (Figure 3D). In summary, the results showed that the character of mtDNA may indicate the outcome of CRC.

### MT-ND1 content in cfDNA of CRC patients

**FIGURE 4:**
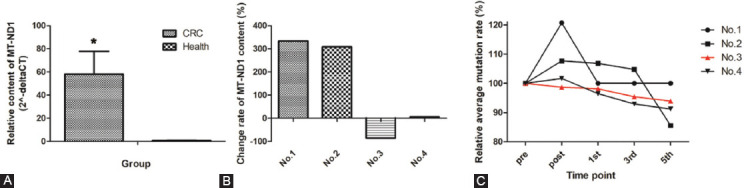
Comparison of MT-ND1 levels in CRC patients and healthy individuals (A) Comparison of MT-ND1 levels in CRC patients and healthy individuals. (B) Comparison of MT-ND1 level changes in plasma of longitudinal cases. (C) Comparison of MT-ND1 mutational load changes in plasma of longitudinal cases.

Four CRC patients and four healthy individuals were included. The MT-ND1 content in cfDNA was compared in the two groups. We found that the content of MT-ND1 in the plasma of CRC patients was larger than that of healthy individuals (Figure 4A). In the four longitudinal CRC cases, the MT-ND1 content in the plasma of No.3 patient dramatically decreased at the fifth chemotherapy compared to the MT-ND1 content before the operation, which had not been observed in the other three CRC patients (Figure 4B).

The dynamic changes of MT-ND1 mutational load in longitudinal cases were observed (Figure 4C). Based on the mutation status of MT-ND1 for CRC patients, we had found four hotspots of MT-ND1. Here, we would like to find out the correlation between the MT-ND1 mutational load and the prognosis of CRC. The mutational status of the four CRC patients before the surgery was shown in Table S2. We found that most of the hotspots had been seen in the plasma of these CRC patients. Three CRC patients contained the hotspot of m.3970 C>T, which seemed to confirm the result of Table 7. However, we found that there was no correlation between the number of hotspots and the prognosis of CRC. Then we evaluated the dynamic changes of each hotspot in all the treatment. The result showed that the mutational ratio decreased before the fifth chemotherapy in both low and high abundance mutations, but the mutational ratios at the other timepoints were irregular (Figure S1). Interestingly, we found the mutation rate continuously decreased only in No.3 patient with a good prognosis (Figure S1). Meanwhile, as showed in Figure 3C, the MT-ND1 content presented the similar tendency. The data demonstrated that a high mutational load of MT-ND1 may be associated with CRC relapse.

### The dynamic changes of CEA, CA72-4 and CA19-9 in plasma of longitudinal cases

In the past decades, serum CEA, CA19-9 and CA72-4 were used for CRC diagnosis and postoperative monitoring [12,13]. We analyzed the levels of CEA, CA72-4 and CA19-9 in the treatment. The result of Table S3 showed the dynamic changes of CEA, CA72-4 and CA19-9 in the treatment. However, these results indicated that there was no relationship between the dynamic changes of conventional tumor biomarkers and the prognosis of CRC. Therefore, we thought that it was limited to use the traditional biomarkers for early prognosis. Effective biomarkers for the early diagnosis and monitoring of cancer are urgently needed.

## DISCUSSION

CRC is the third most common fatal cancer worldwide. Recent studies suggest CRC encompasses an accumulation of genetic and epigenetic aberrances, which aggregate to drive the essential pathways of CRC initiation and progression along a multistep carcinogenic process [14-18]. Genetic and epigenetic alterations of CRC, including somatic mutation, chromosomal instability (CIN), microsatellite instability (MSI), and the CpG island methylator phenotype (CIMP), all contribute to the elevation of CRC [19-21].

Effective biomarkers for the early diagnosis and monitoring of cancer are urgently needed. Although CEA, CA19-9 and CA72-4 have been used in cancer diagnosis and prognosis for many years, researchers realize the limited sensitivity and try to find some more sensitive targets, including molecular biomarker, antigenic marker and combined muti-biomarkers [22-26]. The results in our study also showed that CA19-9 and CA72-4 demonstrated negative in all the patients before surgery, and one CRC patient showed no positive factor among the three biomarkers. The limited sensitivity forces us to depend on positron emission tomography (PET), computer tomography (CT) and double-balloon enteroscopy [27]. Identification of molecular biomarkers could improve the diagnosis and prognosis of CRC. CfDNA detection is widely used in early diagnosis and follow-up monitoring in cancer clinical research [28-31]. Plasma circulating cfDNA is known as a useful tool for accurate grading of treatment response in cancer patients, providing a better perspective for disease status and more treatment options [32]. Besides the area of cancer, other application areas include prenatal diagnosis, organ transplant, aging and so on [33-37]. Due to the low concentration of cfDNA in plasma, the requirement for sufficient DNA for molecular analysis is difficult. However, the copy number of mtDNA is much higher than the copy number of nDNA in cfDNA. Quantification of mitochondrial DNA in plasma or serum samples has been used as a valuable tool for diagnosis and prognosis in clinical research. A few reports showed that the content of mitochondrial DNA in plasma and serum was related to prognosis in cancer patients [38-41]. In our study, we focused on both states of the cfDNA and cell-free mtDNA, and we found that high content of cell-free mtDNA may indicate the relapse of CRC, while the content of cfDNA, the completeness of cfDNA and the completeness of mtDNA had no obvious association with the outcome of CRC. In CRC, T4216C (Y304H), T3394C (Y30H), C3497T (A64V), 3565_3566insC (Frame shift) were detected in some cohorts, and found to be associated with carcinogenesis and the progression of CRC [42-47]. In the MITOMAP database, we found that the mutations of this cohort, including m.3606 A>G, m.3970 C>T and m.4071 C>T, had been reported previously, while the mutation of m.4086 C>T was new.

Mitochondrial metabolism alteration is a hallmark of cancer and it is often accompanied by the excessive formation of reactive oxygen species (ROS). ROS potentially damaged nDNA, mtDNA, lipoproteins and cell membranes [48-50]. Meanwhile, the accumulation of mtDNA mutations affects various components of the electron transport chain (ETC), leading to oxidative damage, causes energy consumption and increases ROS production with a vicious cycle [51,52]. Mutations in mitochondrial genes encoding complex I were observed to be associated with shorter relapse-free survival (RFS) [53]. MT-ND1 was the core mitochondrial-encoded subunit of mitochondrial respiratory complex I. Besides cancer, MT-ND1 is also associated with mitochondrial encephalopathy, lactic acidosis, and stroke-like episodes (MELAS) and optic nerve disease. Its related pathways include respiratory electron transport, ATP synthesis by chemiosmotic coupling, and heat production by uncoupling proteins and GABAergic synapses. Some researchers found there was a significant association between the presence of MT-ND1 mutations and the postoperative recurrence of localized cancer [54]. However, the research object should not be limited to tumor tissues. In our study, we found that circulating cell-free mtDNA generally presented high concordance with tumor tissues in the experiment *in vivo*. Therefore, we focused on whether there was an association between the character of cell-free MT-ND1 in plasma and the prognosis of CRC. Interestingly, at the time point of the fifth chemotherapy, the MT-ND1 content of the patient with a good prognosis was significantly lower than the MT-ND1 content before surgery. However, the MT-ND1 content of the patient with recurrence was significantly higher than the MT-ND1 content before surgery. Although the mutational burden decreased at the fifth chemotherapy in all the patients, the mutational burden continuously decreased only in the patient with a good prognosis. The result may indicate that a high mutational load of MT-ND1 may be associated with CRC relapse. We may improve the ability for the pharmacodynamic evaluation of chemotherapy and early detection of CRC recurrence.

Several limitations remained in our research. Firstly, the number of samples for longitudinal cases was limited. Secondly, the monitoring period was not long enough. Thirdly, the role of these mutations in response to chemotherapy has not been clarified. In the future study, we will compare some CRC cell lines which harbor these mutations with those without mutations in their response to chemotherapy.

## CONCLUSIONS

In summary, the present study demonstrated that the MT-ND1 content in CRC patients was significantly higher than the MT-ND1 content in healthy individuals. Higher content and mutational load of circulating MT-ND1 indicated poor outcome in CRC. In conclusion, circulating MT-ND1 variants may become a highly versatile tool for diagnosing and monitoring colorectal cancer.

## References

[1] Siegel RL, Miller KD, Jemal A (2019). Cancer statistics, 2019. CA Cancer J Clin.

[2] Hofmann JN, Hosgood HD, Liu CS, Chow WH, Shuch B, Cheng WL (2014). A nested case-control study of leukocyte mitochondrial DNA copy number and renal cell carcinoma in the Prostate, Lung, Colorectal and Ovarian Cancer Screening Trial. Carcinogenesis.

[3] Chatterjee A, Dasgupta S, Sidransky D (2011). Mitochondrial subversion in cancer. Cancer Prev Res (Phila).

[4] Lan Q, Lim U, Liu CS, Weinstein SJ, Chanock S, Bonner MR (2008). A prospective study of mitochondrial DNA copy number and risk of non-Hodgkin lymphoma. Blood.

[5] Hosgood HD, Liu CS, Rothman N, Weinstein SJ, Bonner MR, Shen M (2010). Mitochondrial DNA copy number and lung cancer risk in a prospective cohort study. Carcinogenesis.

[6] Lynch SM, Weinstein SJ, Virtamo J, Lan Q, Liu CS, Cheng WL (2011). Mitochondrial DNA copy number and pancreatic cancer in the alpha-tocopherol beta-carotene cancer prevention study. Cancer Prev Res (Phila).

[7] Brandt U (2006). Energy converting NADH:quinone oxidoreductase (complex I). Annu Rev Biochem.

[8] Beckman KB, Ames BN (1997). Oxidative decay of DNA. J Biol Chem.

[9] Yusnita Y, Norsiah MD, Rahman AJ (2010). Mutations in mitochondrial NADH dehydrogenase subunit 1 (mtND1) gene in colorectal carcinoma. Malays J Pathol.

[10] Ye W, Tang X, Liu C, Wen C, Li W, Lyu J (2017). Accurate quantitation of circulating cell-free mitochondrial DNA in plasma by droplet digital PCR. Anal Bioanal Chem.

[11] Ju YS, Alexandrov LB, Gerstung M, Martincorena I, Campbell PJ (2014). Origins and functional consequences of somatic mitochondrial DNA mutations in human cancer. eLife.

[12] Gao Y, Wang J, Zhou Y, Sheng S, Qian SY, Huo X (2018). Evaluation of Serum CEA, CA19-9 CA72-4 CA125 and Ferritin as Diagnostic Markers and Factors of Clinical Parameters for Colorectal Cancer. Sci Rep.

[13] Marchei P, Cifaldi L, De Benedetto A (1990). Clinical evaluation of the efficacy of the combined determination of serum markers CEA CA 19.9, CA 72.4 as indexes of gastro-intestinal tract neoplasms. G Ital Oncol.

[14] Mahasneh A, Al-Shaheri F, Jamal E (2017). Molecular biomarkers for an early diagnosis, effective treatment and prognosis of colorectal cancer:Current updates. Exp Mol Pathol.

[15] Brenner H, Kloor M, Pox CP (2014). Colorectal cancer. Lancet.

[16] Burki TK (2015). Genetic testing for patients with colorectal cancer. Lancet Oncol.

[17] Coppede F (2014). The role of epigenetics in colorectal cancer. Expert Rev Gastroenterol Hepatol.

[18] Jung G, Hernandez-Illan E, Moreira L, Balaguer F, Goel A (2020). Epigenetics of colorectal cancer:biomarker and therapeutic potential. Nat Rev Gastroenterol Hepatol.

[19] The Lancet Gastroenterology H (2018). Colorectal cancer screening:is earlier better?. Lancet Gastroenterol Hepatol.

[20] Nguyen MT, Weinberg DS (2016). Biomarkers in Colorectal Cancer Screening. J Natl Compr Canc Netw.

[21] Gonzalez-Pons M, Cruz-Correa M (2015). Colorectal Cancer Biomarkers:Where Are We Now?. Biomed Res Int.

[22] Min L, Chen L, Liu S, Yu Y, Guo Q, Li P (2019). Loss of Circulating Exosomal miR-92b is a Novel Biomarker of Colorectal Cancer at Early Stage. Int J Med Sci.

[23] Dastmalchi N, Safaralizadeh R, Nargesi MM (2019). LncRNAs:Potential Novel Prognostic and Diagnostic Biomarkers in Colorectal Cancer. Curr Med Chem.

[24] Yan S, Han B, Gao S, Wang X, Wang Z, Wang F (2017). Exosome-encapsulated microRNAs as circulating biomarkers for colorectal cancer. Oncotarget.

[25] Xue Y, Yu F, Yan D, Cui F, Tang H, Wang X (2014). Zinc-alpha-2-glycoprotein:a candidate biomarker for colon cancer diagnosis in Chinese population. Int J Mol Sci.

[26] Pan JH, Zhou H, Cooper L, Huang JL, Zhu SB, Zhao XX (2019). LAYN Is a Prognostic Biomarker and Correlated With Immune Infiltrates in Gastric and Colon Cancers. Front Immunol.

[27] Patel K, Hadar N, Lee J, Siegel BA, Hillner BE, Lau J (2013). The lack of evidence for PET or PET/CT surveillance of patients with treated lymphoma, colorectal cancer, and head and neck cancer:a systematic review. J Nucl Med.

[28] Yamamoto Y, Uemura M, Fujita M, Maejima K, Koh Y, Matsushita M (2019). Clinical significance of the mutational landscape and fragmentation of circulating tumor DNA in renal cell carcinoma. Cancer Sci.

[29] Lin LH, Chang KW, Kao SY, Cheng HW, Liu CJ (2018). Increased Plasma Circulating Cell-Free DNA Could Be a Potential Marker for Oral Cancer. Int J Mol Sci.

[30] Yamamoto Y, Uemura M, Nakano K, Hayashi Y, Wang C, Ishizuya Y (2018). Increased level and fragmentation of plasma circulating cell-free DNA are diagnostic and prognostic markers for renal cell carcinoma. Oncotarget.

[31] Gao YJ, He YJ, Yang ZL, Shao HY, Zuo Y, Bai Y (2010). Increased integrity of circulating cell-free DNA in plasma of patients with acute leukemia. Clin Chem Lab Med.

[32] Calapre L, Warburton L, Millward M, Gray ES (2019). Circulating tumour DNA (ctDNA) as a biomarker in metachronous melanoma and colorectal cancer- a case report. BMC Cancer.

[33] Valderramos SG, Rao RR, Scibetta EW, Silverman NS, Han CS, Platt LD (2016). Cell-free DNA screening in clinical practice:abnormal autosomal aneuploidy and microdeletion results. Am J Obstet Gynecol.

[34] Burnham P, Khush K, De Vlaminck I (2017). Myriad Applications of Circulating Cell-Free DNA in Precision Organ Transplant Monitoring. Ann Am Thorac Soc.

[35] Le Conte G, Letourneau A, Jani J, Kleinfinger P, Lohmann L, Costa JM (2018). Cell-free fetal DNA analysis in maternal plasma as screening test for trisomies 21 18 and 13 in twin pregnancy. Ultrasound Obstet Gynecol.

[36] De Vlaminck I, Martin L, Kertesz M, Patel K, Kowarsky M, Strehl C (2015). Noninvasive monitoring of infection and rejection after lung transplantation. Proc Natl Acad Sci U S A.

[37] Teo YV, Capri M, Morsiani C, Faria AMC, Franceschi C (2019). Cell-free DNA as a biomarker of aging. Aging Cell.

[38] Koochekpour S, Marlowe T, Singh KK, Attwood K, Chandra D (2013). Reduced mitochondrial DNA content associates with poor prognosis of prostate cancer in African American men. PLoS One.

[39] Mehra N, Penning M, Maas J, van Daal N, Giles RH, Voest EE (2007). Circulating mitochondrial nucleic acids have prognostic value for survival in patients with advanced prostate cancer. Clin Cancer Res.

[40] Meng X, Schwarzenbach H, Yang Y, Muller V, Li N, Tian D (2019). Circulating Mitochondrial DNA is Linked to Progression and Prognosis of Epithelial Ovarian Cancer. Transl Oncol.

[41] Lee JH, Hwang I, Kang YN, Choi IJ, Kim DK (2015). Genetic characteristics of mitochondrial DNA was associated with colorectal carcinogenesis and its prognosis. PLoS One.

[42] Akouchekian M, Houshmand M, Akbari MH, Kamalidehghan B, Dehghan M (2011). Analysis of mitochondrial ND1 gene in human colorectal cancer. J Res Med Sci.

[43] Kassem AM, El-Guendy N, Tantawy M, Abdelhady H, El-Ghor A, Abdel Wahab AH (2011). Mutational hotspots in the mitochondrial D-loop region of cancerous and precancerous colorectal lesions in Egyptian patients. DNA Cell Biol.

[44] Yusnita Y, Norsiah MD, Rahman AJ (2010). Mutations in mitochondrial NADH dehydrogenase subunit 1 (mtND1) gene in colorectal carcinoma. Malays J Pathol.

[45] Koshikawa N, Akimoto M, Hayashi JI, Nagase H, Takenaga K (2017). Association of predicted pathogenic mutations in mitochondrial ND genes with distant metastasis in NSCLC and colon cancer. Sci Rep.

[46] Weerts MJA, Timmermans EC, van de Stolpe A, Vossen R, Anvar SY, Foekens JA, Sleijfer S, Martens JWM (2018). Tumor-specific mitochondrial DNA variants are rarely detected in cell-free DNA. Neoplasia.

[47] Pinheiro M, Veiga I, Pinto C, Afonso L, Sousa O, Fragoso M, Santos L, Lopes P, Pais I, Lopes C, Teixeira MR (2009). Mitochondrial genome alterations in rectal and sigmoid carcinomas. Cancer Lett.

[48] Multhoff G, Molls M, Radons J (2011). Chronic inflammation in cancer development. Front Immunol.

[49] Piotrowski I, Kulcenty K, Suchorska W (2020). Interplay between inflammation and cancer. Rep Pract Oncol Radiother.

[50] Lichtenstern CR, Ngu RK, Shalapour S, Karin M (2020). Immunotherapy, Inflammation and Colorectal Cancer Cells.

[51] Beal MF (2007). Mitochondria and neurodegeneration. Novartis Found Symp.

[52] Iommarini L, Ghelli A, Gasparre G, Porcelli AM (2017). Mitochondrial metabolism and energy sensing in tumor progression. Biochim Biophys Acta Bioenerg.

[53] Uzawa K, Kasamatsu A, Baba T, Kimura Y, Nakashima D, Higo M (2015). Quantitative detection of circulating tumor-derived mitochondrial NADH subunit variants as a potential prognostic biomarker for oral cancer. Int J Oncol.

[54] Kim H, Komiyama T, Inomoto C, Kamiguchi H, Kajiwara H, Kobayashi H (2016). Mutations in the Mitochondrial ND1 Gene Are Associated with Postoperative Prognosis of Localized Renal Cell Carcinoma. Int J Mol Sci.

